# Effect of the CRADLE vital signs alert device intervention on referrals for obstetric haemorrhage in low-middle income countries: a secondary analysis of a stepped- wedge cluster-randomised control trial

**DOI:** 10.1186/s12884-021-03796-4

**Published:** 2021-04-21

**Authors:** Lucie Giblin, Nicola Vousden, Hannah Nathan, Francis Gidiri, Shivaprasad Goudar, Umesh Charantimath, Jane Sandall, Paul T. Seed, Lucy C. Chappell, Andrew H. Shennan

**Affiliations:** 1 Department of Women and Children’s Health, School of Life Course Sciences, Faculty of Life Sciences and Medicine, King’s College London, London, SE1 7EH UK; 2 Department of Obstetrics and Gynaecology, College of Health Sciences, University of Zimbabwe, Harare, Zimbabwe; 3 Women’s and Children’s Health Research Unit, KLE Academy of Higher Education and Research, Jawaharlal Nehru Medical College, Belgaum, Karnataka India

**Keywords:** Referral, Shock index, Obstetric haemorrhage, Bleeding, Triage, Resource allocation

## Abstract

**Background:**

Obstetric haemorrhage is the leading cause of maternal death worldwide, 99% of which occur in low and middle income countries. The majority of deaths and adverse events are associated with delays in identifying compromise and escalating care. Management of severely compromised pregnant women may require transfer to tertiary centres for specialised treatment, therefore early recognition is vital for efficient management. The CRADLE vital signs alert device accurately measures blood pressure and heart rate, calculates the shock index (heart rate divided by systolic blood pressure) and alerts the user to compromise through a traffic light system reflecting previously validated shock index thresholds.

**Methods:**

This is a planned secondary analysis of data from the CRADLE-3 trial from ten clusters across Africa, India and Haiti where the device and training package were randomly introduced. Referral data were prospectively collected for a 4-week period before, and a 4-week period 3 months after implementation. Referrals from primary or secondary care facilities to higher level care for any cause were recorded. The denominator was the number of women seen for maternity care in these facilities.

**Results:**

Between April 1 2016 and Nov 30th, 2017 536,223 women attended maternity care facilities. Overall, 3.7% (*n* = 2784/74,828) of women seen in peripheral maternity facilities were referred to higher level care in the control period compared to 4.4% (*n* = 3212/73,371) in the intervention period (OR 0.89; 0.39–2.05) (data for nine sites that were able to collect denominator). Of these 0.29% (*n* = 212) pre-intervention and 0.16% (*n* = 120) post-intervention were referred to higher-level facilities for maternal haemorrhage. Although overall referrals did not significantly reduce there was a significant reduction in referrals for obstetric haemorrhage (OR 0.56 (0.39–0.65) following introduction of the device with homogeneity (i-squared 26.1) between sites. There was no increase in any bleeding-related morbidity (maternal death or emergency hysterectomy).

**Conclusions:**

Referrals for obstetric haemorrhage reduced following implementation of the CRADLE Vital Signs Alert Device, occurring without an increase in maternal death or emergency hysterectomy. This demonstrates the potential benefit of shock index in management pathways for obstetric haemorrhage and targeting limited resources in low- middle- income settings.

**Trial registration:**

This study is registered with the ISRCTN registry, number ISRCTN41244132 (02/02/2016).

## Background

We have recently demonstrated obstetric haemorrhage to be the cause of 36% of maternal death in urban areas of low-middle income countries [1]. Haemorrhage is also a significant contributor to long-term disability, prolonged recovery and organ dysfunction associated with shock and remains a major challenge to health systems worldwide [2]. A woman dies due to postpartum haemorrhage (PPH) approximately every 4 min [3]. The majority of maternal deaths from bleeding are preventable through early recognition of deterioration, allowing prompt management or referral for specialist care. Effective triage means that scarce resources can be targeted to those at greatest risk of death and other complications [4]. There is substantial intercountry variation in incidence of severe postpartum haemorrhage and this may result from differing health care systems and referral pathways.

It has been suggested that an efficient strategy for low-resource countries to reduce maternal and neonatal complications is placing skilled birth attendants at health centres with referral capacity, and therefore institutional delivery at community (primary care) and district health centres (secondary care) is promoted [5]. The framework of the three delays conceptualises that maternal outcome is most adversely affected by factors that delay decisions to seeking care, arrival at the health facility or provision of adequate care [6, 7]. Referral and patient transfer introduce potential delay to necessary care. Efficient decision making within the referral process could limit delays to accessing care and ensure the correct patients are referred promptly.

Vital signs are used to assess haemodynamic state and detect deterioration in hypovolemic shock. The CRADLE (Community blood pressure monitoring in Rural Africa & Asia: Detection of underLying pre-Eclampsia and shock) Vital Signs Alert device (CRADLE VSA) is a semi-automated vital signs measurement device developed specifically for use in low-resource settings. Unlike most other commercially available devices, it is low cost, accurate and has been specifically validated for use in pregnancy, pre-eclampsia and shock [8–13]. It measures a patient’s blood pressure and heart rate, calculating the shock index (heart rate divided by systolic blood pressure) and displays a traffic light early warning system based on shock index thresholds. We have validated these thresholds (red (1.7) and amber (0.9)) in low- and middle- income countries in women with postpartum haemorrhage and have demonstrated predictive value with adverse outcomes (admission to high dependency units, blood transfusion over 4 units and maternal death) [10–14]. Shock index compares favourably to conventional vital signs in consistently predicting risk of adverse clinical outcomes in women with post-partum haemorrhage [15].

Integrated into the CRADLE VSA device, shock index could be a valuable tool for risk stratifying patients. Its simplicity is important where routine clinical tasks are undertaken by health care workers, students, or volunteers, and where community health workers are the vital link to emergency services for unwell patients [5, 16]. We anticipate that the CRADLE VSA device identifies patients requiring referral for haemorrhagic shock and that this will be of benefit in low-income countries where decisions frequently fall to untrained health workers [9, 17–19]. However, improved identification of at-risk women must be balanced against overall referral rates and clinical outcomes given the limited resources available.

The CRADLE-3 trial was a pragmatic, stepped-wedge, cluster-randomised controlled trial to evaluate the effect of the CRADLE VSA device on maternal mortality and morbidity in low-resource settings. The results demonstrated that there was a reduction in emergency hysterectomy, a pre-defined secondary outcome, as might be anticipated with earlier recognition of haemorrhagic shock. However, after adjustment for between-centre variability, which was greater than anticipated, there was insignificant evidence to demonstrate the efficacy of the intervention on the primary composite outcome (at least one of eclampsia, hysterectomy and maternal death). This secondary analysis of our recent CRADLE-3 trial evaluates the impact of using the CRADLE VSA in management of referrals for bleeding women in multiple low-resource settings.

## Method

This is a planned secondary analysis of the CRADLE 3 trial; a pragmatic, step-wedge, cluster- randomised control trial that evaluated the CRADLE VSA intervention (CVSA device and training package) in low resource settings [1].

### Study design

The CRADLE 3 intervention consisted of implementing the CRADLE VSA device and associated training package in routine community and hospital maternity care in low-resource settings. Prior to implementation of the intervention package, management was based on local guidelines and assessment of patients used varying medical devices, and this was used as a control. The trial was carried out across ten clusters over eight countries, including Addis Ababa (Ethiopia), Cap Haitien (Haiti), Freetown (Sierra Leone), Harare (Zimbabwe), Gokak (India), Kampala and Mbale (Uganda), Lusaka and Ndola (Zambia), and Zomba and the Southern Region (Malawi). Each cluster included at least one urban or peri-urban secondary or tertiary facility and multiple peripheral hospitals that referred to the region’s central hospital. In total 286 facilities and 536,223 deliveries were included in the CRADLE intervention between April 1 2016 and November 30 2017.

Clusters crossed over from the control to the intervention at a randomly allocated timepoint, at 2 monthly intervals. At this randomly selected timepoint, all existing devices were replaced with the CRADLE VSA and health care providers at the facility were provided with access to the device and training package. Prior to intervention, management was based on local guidelines and assessment of patients used varying medical devices, and this was used as a control. Ethics approval was granted by the King’s College London (UK) Research Ethics Subcommittee (LRS-14/15–1484) and in all countries before the start of the trial. Institutional-level consent on behalf of the cluster was obtained. In total 3868 devices were delivered to 286 facilities.

### Participants

All women identified as pregnant or up to 42 days postnatal presenting to the facility were eligible to be exposed to the intervention. There were no exclusion criteria.

### Randomisation

The randomisation was the cluster. A computer-generated randomly allocated sequence run by the CRADLE statistician determined the order in which the clusters received the intervention. All clusters were masked to the order of implementation until 2 months before the intervention. Because of the nature of the intervention the trial was not masked.

### Procedures

At each randomly allocated date, the training package was delivered to health workers in each facility by interactive group sessions. Existing equipment for vital signs observations was replaced with the CRADLE device unless specific function were required (e.g. cyclical BP monitoring in HDU).

The proportion of women referred from periphery facilities to higher-level care was collected from a 4-week period before and another 4-week period repeated 3 months post-implementation. Referrals were either counted from referral registers and compared to number of patients seen in antenatal clinic or admitted to ward, or data was documented prospectively as patients were referred. It was not possible to collect accurate denominator referral data in one large site with multiple referral areas (Kampala) therefore this area was not included.

Maternity staffing levels and access to essential treatment (intensive care beds, capacity for blood transfusion) were also assessed at each facility and recorded throughout the trial period. Major changes to infrastructure, patient payment requirements, or environmental conditions were systematically evaluated each month in each site. The trial ended after 20 months as planned. Reason for referral was documented as infection, bleeding, high blood pressure, labour or other (which included anaemia, malaria, early pregnancy complications).

### Outcomes

The primary outcome of the CRADLE-3 trial was a composite of at least one of eclampsia, hysterectomy or maternal death. In this analysis we reviewed the number of patients referred from primary to secondary or tertiary higher-level care for bleeding pre- and post- intervention. We report outcomes from the CRADLE-3 trial related to haemorrhage i.e. death from obstetric haemorrhage or emergency hysterectomy due to obstetric haemorrhage.

### Statistical analysis

We evaluated the effect of implementation on referrals for bleeding through a planned secondary analysis of the CRADLE-3 data. Odds ratios were calculated for each centre, comparing event rates pre- and post- CRADLE intervention. As there was considerable heterogeneity, random effects meta-analysis was used throughout [20].

For evaluation of outcome the CRADLE-3 trial reports the bent stick analysis. This achieves great stability to the trend-and-step pattern originally proposed in the CRADLE 3 trial, because it allows for separate linear trends in each cluster before and after intervention.

Statistical analyses used Stata, version 14.2 (by PTS). This study is registered with the ISRCTN registry, number ISRCTN41244132.

## Results

Between April 1 2016 and Nov 30th, 2017 536,223 women attended maternity care facilities. In our previous paper, we reported that 2784 (3.7%) of 74,828 women seen in peripheral maternity facilities were referred in the pre-intervention period compared with 3212 (4.4%) of 73,371 women in the intervention period (adjusted OR 0.89, 95% CI 0.39–2.05; data from the Mulago (Kampala) cluster were excluded because the site was unable to collect the denominator) [1]. The majority of sites demonstrated a small but significant decrease in referrals with a single site (Gokak) demonstrating a 16-fold increase. (Note that once allowed for grouping the effects have been reversed from a rise in referrals to an odds ratio < 1. This can be likened to the Simpson-Yule paradox phenomenon in statistics, in which a trend disappears or reverses when groups are combined and may relate to the use of random-effects meta-analysis. This result regardless is insignificant and should not be over-interpreted.)

**Fig. 1:**
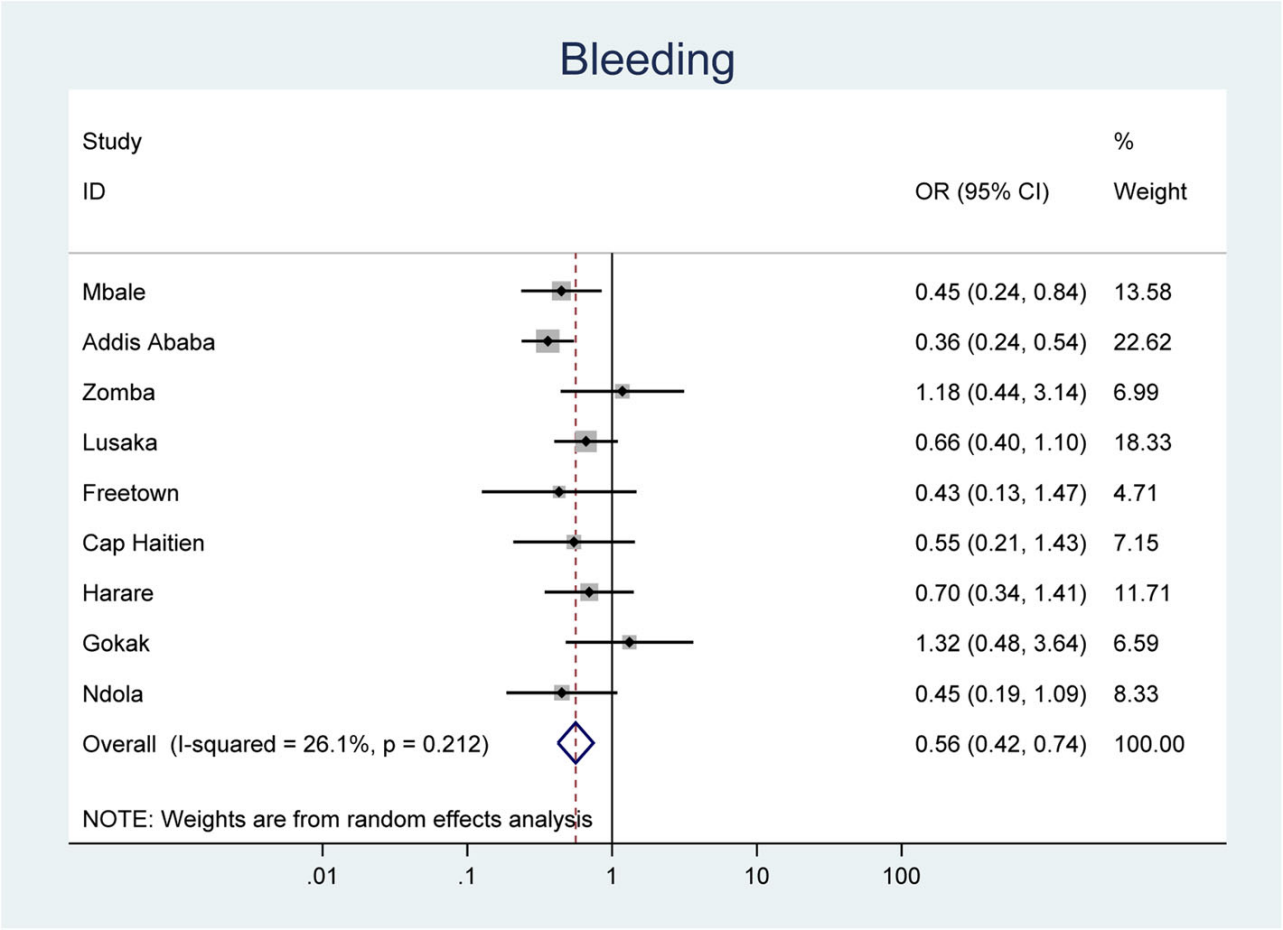
Forest plot displaying random effects meta-analysis of referrals for bleeding from all sites

By disaggregating these data by indication for referral, we have shown that referrals for haemorrhage significantly reduced from 212 women (0.29%) to 120 women (0.16%) (OR 0.56 95% CI 0.42–0.74 *p* = 0.212) following intervention as shown in Fig. 1. This was not associated with any significant change in death from haemorrhage, as shown in our previous trial report [1], using either the trend and step comparison (adjusted OR 0·86; 95% CI 0·56–1·33) or bent stick comparison (adjusted OR 0.56; 95% CI 0.29–1.0), and with no change in emergency hysterectomy for postpartum haemorrhage (trend and step comparison: adjusted OR 1.23; 95% CI 0·72–2·10; bent stick comparison: adjusted OR 0.45 95% CI 0.11–1.09). There was no change in the proportion of deaths from all causes that occurred in the community compared to in health care facilities.

## Discussion

These findings indicate the CRADLE VSA training package is a safe intervention that can reduce strain on tertiary services. We can speculate that the documented benefit of shock index in diagnosing haemorrhagic shock may have improved prompt management thus reducing rates of massive haemorrhage requiring referral. Shock index bases risk assessment of the patient on haemodynamic observations as opposed to visual estimation of loss or late signs such as collapse. The CRADLE VSA and training package highlighted evidence-based treatment protocols guided by shock index thresholds, which may have improved management and mitigated patient compromise. This analysis supports existing evidence that the CRADLE VSA intervention improves capacity to make clinical decisions, escalate care and make appropriate referrals [21].

A problem encountered in low-resource settings is referral of the moribund patient, and therefore recognition around when to treat versus when to refer is essential. CRADLE-3 data demonstrates that proportion of deaths in each level of care remained similar pre- and post- intervention. This supports the findings that referrals were sensible, and patients were not simply referred later after attempts to treat locally.

Predefined blood loss thresholds are difficult to measure accurately, especially when patient transfer in involved, and do not accurately represent severity of outcome. Shock index can be used to identify the early stages of haemodynamic compromise and trigger intervention in those that need it. Conversely its use can avoid unnecessary intervention in patients that cope well with blood loss if they remain clinically stable. A normal shock index in spite of haemorrhage (which would normally prompt referral based on visual estimation of blood loss) may reassure clinicians and allow patients to be treated locally preventing unnecessary referral. Use of shock index bases decisions on a patients haemodynamic state as opposed to arbitrary values of blood loss which may not be clinically useful. The CRADLE VSA is more accurate (especially in low blood pressure associated with shock), durable, and easier-to-use than commonly used machines, thus improving vital sign observation.

Emergency transport is often scarce in these environments and shared with other specialties. Transfer can be on foot or public transport, incurring significant time delay and cost to patients or their families. Reducing referrals and targeting resources to those most at need is likely to be beneficial. Reducing unnecessary use of scarce ambulances will free up availability for use in any subsequent adverse event. Future research needs to equate the safety of shock index in this setting particularly as a rule-out test for referral. Our data is supported by previous observations that a green light (representing SI < 0.9) is rarely associated with significant morbidity in women with post-partum haemorrhage [22].

### Strengths and limitations of this study

The strengths of these data are the multiple countries and multiple levels of health care facilities involved, as well as the rigorous data collection methods. The CRADLE-3 trial was multicentre, randomised, cluster controlled and evaluated the device across over 500,000 deliveries.

Data on all patients attending the facilities during the period of analysis was not collected therefore number of bleeding patients referred cannot be presented as a proportion of all bleeding patients who presented (denominator was patients presenting for all causes). Cause and timing of bleeding was not documented and therefore not assessed. Further research into the effect of the CRADLE VSA on rates of haemorrhage and time to management in peripheral units would aid in this evaluation.

The outcomes we use in support of the safety of this reduction in referrals are limited to emergency hysterectomy and death only. Potential adverse outcomes associated with reduced referrals include near-misses. We did not assess near misses that may have occurred as a result of the intervention, but overall the trial was associated with no increase in morbidity or mortality related to haemorrhage and the best estimates show a non-significant reduction. We were unable to control for time trends, however it remains unlikely, given the provision of other interventions remained constant, that the substantive reduction in referrals can be accounted for by other causes. There is a chance that referral data may have been collected differently following CVSA training package, thus affecting data collection. To mitigate this risk, health care professionals were encouraged to report in the same manner and a trained research teams collected the data.

Although the introduction of the CRADLE VSA was not associated with a significant change in referral patterns overall, there was significant homogeneity in this data. One site (Gokak, India) was an outlier with a substantially high referral rate that increased 15 fold following intervention (1.12% to 15.3%). On interrogation we believe this was related to shock index > 0.9 being triggered by severe anaemia, a condition highly prevalent in Karnataka. This is a subject of further research at present. When this outlier is removed, overall referrals were significantly reduced by the CVSA for all causes (OR 0.62 (0.43–0.90 *p* = 0), but none as great as those for haemorrhage. During the trial period there was a 9% reduction in mortality in absolute terms pre- and post- intervention [1].

## Conclusion

Implementation of the CVSA and training reduced referrals to higher level care for bleeding women without significantly increasing death or emergency hysterectomy. Overall referrals did not increase significantly.

Poor outcomes following obstetric haemorrhage have been attributed to delayed treatment, inaccurate estimation of blood loss, absence of treatment protocols, poor communication among the treating teams, and inadequate organisational support [23, 24]. We propose the use of the shock index as an accurate and simple observation tool that, when integrated into the CRADLE VSA intervention can improve efficiency in referral and management. This secondary analysis of the CRADLE-3 trial determines that the CRADLE VSA intervention reduces strain on systems from bleeding patients without a negative effect on outcome. Further analysis would be useful in evaluating the use of shock index in reducing time to treatment and reducing poor obstetric outcomes through a prospective in-country analysis and trial of its use in management.

## Data Availability

The dataset from CRADLE-3 is available to appropriate academic parties on request from the Chief Investigator in accordance with the data sharing policies of King’s College London, UK, with input from the Co-investigator where applicable.
